# Severity Profile of COVID-19 in Hospitalized Pediatric Patients

**DOI:** 10.3390/children11101249

**Published:** 2024-10-16

**Authors:** Vânia Chagas da Costa, Ulisses Ramos Montarroyos, Katiuscia Araújo de Miranda Lopes, Ana Célia Oliveira dos Santos

**Affiliations:** 1 Postgraduate Program in Health Sciences, Universidade de Pernambuco, Recife 50100-010, Pernambuco, Brazil; ulisses.montarroyos@upe.br (U.R.M.); ana.oliveira@upe.br (A.C.O.d.S.); 2 School of Nursing Our Lady of Grace, Universidade de Pernambuco, Recife 50100-010, Pernambuco, Brazil; katiuscia.lopes@upe.br

**Keywords:** COVID-19, child, signs and symptoms, risk factors, asthma, severe acute respiratory syndrome

## Abstract

Objective: We aimed to describe the clinical characteristics associated with severity in children hospitalized with COVID-19. Method: This was an epidemiological cohort study conducted in two hospitals, one of which was a reference center for the treatment of COVID-19 cases. Data were collected from the reports generated by the hospital epidemiology centers and the medical records of patients aged between 0 and 14 years with a diagnosis of COVID-19, hospitalized between March 2020 and June 2021. To analyze the association between the clinical profile and severity, the cases were classified as severe (severe and critical) and non-severe (asymptomatic, mild, and moderate). Results: Of the 191 children followed up in the cohort, 73.3% developed the severe form. The percentage of children with oxygen saturation below 95% was 46.6%. In the multivariate analysis, a higher risk of severity was estimated among children with uncontrolled asthma (RR = 13.2), who were overweight or obese (RR = 3.21), who had cough symptoms (RR = 2.72), and those aged under one year (RR = 3.23). Conclusions: This result underscores the need to improve healthcare at every level for children and for the management of asthma and nutrition when considering children with this clinical profile who are diagnosed with COVID-19.

## 1. Introduction

SARS-CoV-2, the coronavirus 2, responsible for the severe acute respiratory syndrome (SARS) and the causative agent of COVID-19, was declared a public health emergency of international concern (PHEIC) by the World Health Organization (WHO) in 2020 [1]. Having been identified in December 2019, this highly contagious virus had spread to over 210 countries by May 2020, infecting approximately 3.8 million people and resulting in around 300,000 deaths [2].

Over the course of the pandemic, more than 772 million people reported having been infected with COVID-19, resulting in a global death toll exceeding 6.9 million. While new case numbers have gradually decreased, leading the WHO to declare an end to the PHEIC in May 2023 [3], the virus has nonetheless continued to claim lives across all age groups.

The SARS-CoV-2 virus primarily affects the lungs but may also affect other organs such as the heart, brain, kidneys, liver, spleen, skin, placenta, and testicles [4]. It is primarily transmitted through inhalation or contact with respiratory droplets expelled by symptomatic infected individuals during coughing and sneezing [4,5,6].

Studies have demonstrated that children infected with SARS-CoV-2 are asymptomatic or present mild symptoms. This can be attributed to the trained immune response derived from vaccines and frequent viral infections, the ability to regenerate the alveolar epithelium, and the simultaneous presence of another virus in the respiratory mucosa competing with SARS-CoV-2, thereby facilitating early control of the COVID-19 infection. Additionally, children also exhibit a lower prevalence of comorbidities such as hypertension, diabetes, and chronic pulmonary disease [1,6].

However, children are not exempt from presenting severe and fatal forms of the disease, such as severe acute respiratory syndrome (SARS) and pediatric inflammatory multisystem syndrome (PIMS). In Brazil, between January and August 2023, there were 3441 reported cases and 84 deaths attributed to SARS associated with COVID-19 in children aged under one year [7].

While severity and mortality from COVID-19 were less common in children and adolescents overall, neonates and infants aged under one year have demonstrated increased vulnerability. The most frequent symptoms observed in these age groups included fever, cough, and difficulty breathing [8,9,10].

To address the pandemic, public health services, including pediatric care, were structured so that those infected with SARS-CoV-2 were isolated in an attempt to curtail its spread within the general population. However, with the implementation of routine testing, cases of infected children emerged even in hospitals that were not COVID-19 referral centers. Upon detection, children exhibiting clinical improvement were discharged for home isolation, whereas those requiring continuous monitoring were transferred to COVID-19 referral hospitals.

Identifying the sociodemographic and clinical characteristics associated with an increased risk of severe SARS-CoV-2 could contribute to a better understanding of how COVID-19 manifests in the child population. This information could optimize the management of severe cases of COVID-19 in children and their care during hospitalization. This study aimed to describe the clinical characteristics associated with disease severity among children hospitalized with COVID-19.

## 2. Materials and Methods

Type of Study: This is a cohort epidemiological study.

Study Site: The study was conducted in two hospitals in the state of Pernambuco, Brazil. The Hospital Universitário Oswaldo Cruz, affiliated with the Universidade de Pernambuco, serves as a state referral center for the treatment of patients infected with COVID-19. The Hospital de Pediatria Helena Moura, which provides clinical, accident, and emergency pediatric care, is linked to the Recife Municipal Health Network, although it was not a COVID-19 referral center during the pandemic.

Participants: The studied population consisted of hospitalized pediatric patients aged from zero to 14 years who were diagnosed with COVID-19 based on laboratory criteria and had a positive RT-PCR (reverse-transcriptase polymerase chain reaction) test for SARS-CoV-2.

Data Collection: Data was collected from reports generated by the epidemiology centers of the involved hospitals, encompassing all identified cases of COVID-19. Subsequently, hospital inpatient records for the period of March 2020 to June 2021 were reviewed, coinciding with the initiation of SARS-CoV-2 testing in March 2020. Case analysis only continued until June 2021 due to the start of COVID-19 vaccination for the study population, which could have potentially modified clinical characteristics. Patients with comorbidities, with the exception of asthma, were excluded from the study. As the most prevalent chronic non-communicable childhood disease, asthma can necessitate hospitalization depending on its severity and controllability and was initially listed as a risk factor for COVID-19. Other comorbidities were excluded so that the severity of cases would not be confused with a worsening of the underlying disease.

Study Variables and Data Collection Instrument: To collect data from both hospitals, a standardized form was developed containing sociodemographic and clinical variables such as age, sex, weight, symptomatology, diagnostic hypothesis that motivated hospital admission, use of oxygen therapy, ventilatory support, laboratory and imaging exams, drug therapy, admission to the pediatric intensive care unit (PICU), and length of hospital stay.

The cases of COVID-19 were classified according to the criteria established in the definitions of the Brazilian Ministry of Health: Asymptomatic case—characterized by a positive laboratory test for COVID-19 and the absence of symptoms; Mild case—characterized by the presence of non-specific symptoms, such as cough, sore throat or runny nose, followed or not by anosmia, ageusia, diarrhea, abdominal pain, fever, chills, myalgia, fatigue, and/or headache; Moderate case—characterized by common symptoms that can include mild signs of the disease, such as a persistent cough and daily persistent fever, and signs of the progressive worsening of another symptom related to COVID-19 (adynamia, prostration, hyporexia, or diarrhea) in addition to the presence of pneumonia without signs or symptoms of severity; Severe case—characterized by severe acute respiratory syndrome (SARS) flu syndrome that presents as dyspnea/respiratory discomfort or persistent pressure in the chest, oxygen saturation less than 95% in ambient air, or a bluish coloring of the lips or face. For children, the main symptoms include tachypnea (greater than or equal to 70 rpm for children under 1 year and greater than or equal to 50 rpm for children over 1 year), hypoxemia, respiratory discomfort, altered consciousness, dehydration, myocardial injury, elevated liver enzymes, coagulation dysfunction, rhabdomyolysis, central cyanosis or SpO2 < 90–92% at rest and in ambient air, lethargy, seizures, and difficulty feeding/refusing food; and Critical case—characterized by sepsis, acute respiratory distress syndrome, severe respiratory failure, multiple organ dysfunction, severe pneumonia, a need for respiratory support, and hospitalization in intensive care units.

Asthma comorbidity was defined by the continuous/daily use of two inhaled control/preventive medications, omalizumab or oral corticosteroids, or a history of asthma-related hospital admission within the past twelve months, which is indicative of uncontrolled and severe asthma.

Treatment and Data Analysis: Anthropometric indicators were calculated using the World Health Organization (WHO) Anthro software version 3.2.2 and AnthroPlus software version 1.0.4 developed to facilitate the application of WHO growth reference curves for children aged zero to five years (Anthro) and five to nineteen years (Anthro Plus). The assessed children were categorized based on their nutritional status as either presenting adequate weight or being overweight/obese.

MS Office Excel for Mac software version 16.89.1 was employed for the database management of both hospitals. For statistical analysis, asymptomatic, mild, and moderate cases were categorized as non-severe, while severe and critical cases were combined to form the severe category. This classification facilitated the identification of predictor variables associated with disease severity.

Relative frequencies (percentages) and absolute frequencies (n) were presented for the categorical variables. In the association analysis, the severity variable of COVID-19 was related to the previously mentioned independent variables. Associations with a *p*-value < 0.20 were eligible for multiple logistic regression using the stepwise methodology, discarding variables identified as collinear from the model. In the final model of adjusted risks, the association was significant when the *p*-value was <0.05. Data analysis used Stata 14.

## 3. Results

In the referral hospital for COVID-19 treatment, 171 children tested positive for SARS-CoV-2 infection confirmed by RT-PCR, representing 17% of the investigated cases. Of these, 66 children were excluded due to underlying conditions such as oncological diseases, hereditary genetic diseases, genetic syndromes, chronic encephalopathies, congenital heart diseases, and HIV exposure, resulting in a sample of 105 children. At the pediatric hospital, an additional 86 cases of SARS-CoV-2 infection were confirmed through RT-PCR.

The study sample, therefore, consisted of 191 pediatric patients hospitalized with a positive RT-PCR diagnosis for SARS-CoV-2, of which 73.3% were severe cases and 26.7% were non-severe cases. Notably, 46.6% of children experienced oxygen saturation levels below the critical threshold of 95%. Cases of greater severity affected children aged under five years, with the highest incidence observed among infants aged under one year, as presented in Table 1. The median length of hospital stay for the sample was five days, with a minimum of one and a maximum of 140 days. In terms of the outcomes, 99.5% were discharged from hospital, and just one death was observed within the group (0.5%).

In Table 2, when comparing the biological, clinical, and symptomatic characteristics of severe and non-severe cases, it may be observed that severe cases were more frequent in children under one year of age, those who were male, those who were overweight/obese, those with a comorbidity of asthma, and those with signs and symptoms of cough, myalgia, fever, fatigue, diarrhea, and abdominal pain.

After multivariate adjustment, as presented in Table 3, there was an association with the comorbidity of asthma. The association between severity and the child’s nutritional status was borderline. From among the symptoms, cough was associated with severity. Children aged under one year exhibited a 3.2-fold increased risk of developing a severe case when compared to children aged over five years.

In the comparative analysis of data from the referral hospital for COVID-19 and the pediatric hospital, no significant differences were observed, maintaining the associations found in the multivariate analysis.

## 4. Discussion

When analyzing the study sample, we observed a higher rate of hospitalizations due to COVID-19 among children who were aged under five years, male, and classified as severe cases, although in most cases with a favorable outcome. The severity of COVID-19 in hospitalized children was associated with children aged under one year of age, with asthma and an overweight/obese nutritional status as comorbidities and cough as a clinical sign.

In the present study, severe cases represented a larger proportion among those hospitalized with a diagnosis of COVID-19. The severity of these children, predominantly aged under five years, was characterized by oxygen saturation below 95%, respiratory distress, and the requirement for oxygen therapy, including mechanical ventilation and PICU admission. This result aligns with other studies conducted in Brazil [11,12], China [13], the United States [14], and Turkey [15]. Consistent with our results, Dong Y et al. (2020) and Zhang et al. (2023) also reported an association between greater severity and an age group with a higher risk among children aged under one year [13,16].

With regard to sex, no statistically significant difference was observed indicating a greater severity of COVID-19. However, there was a higher proportion of severe cases in males when compared to females in the studied sample. While similar findings have been reported in the literature [12,17], a study in Wuhan, China [18] observed no direct evidence supporting a greater susceptibility of females or males to SARS-CoV-2 infection. Conversely, other studies [19,20,21] have suggested that the X chromosome may offer females protection against severe COVID-19.

This study has highlighted the ongoing debate regarding the role of asthma as a risk factor for severe COVID-19. While an association between the severity of COVID-19 and uncontrolled asthma has been observed, chronic lung diseases, including asthma, have only slightly increased the risk of severe COVID-19 among children. However, some studies have found no significant association between asthma and the severity of COVID-19 [22,23], suggesting that well-controlled asthma does not increase the risk of severe COVID-19.

However, in a scenario of limited access to healthcare, as experienced by the population in this study, asthma was only ever treated during exacerbations necessitating hospitalization. This finding aligns with research that detected an elevated risk of severe COVID-19 among patients requiring oral corticosteroids for asthma control and those hospitalized for severe asthma exacerbations [24].

Asthma manifests as respiratory impairment resulting from a generally uncontrolled, chronic airway inflammation. Symptoms can be exacerbated due to infections by various types of pathogens, including SARS-CoV-2. Asthma control reflects treatment effectiveness in reducing clinical manifestations, while asthma severity correlates with the medication dosage required to achieve control [24].

Because the healthcare system in Brazil, as in other parts of the world, needed to be adapted to confront the rapid spread of COVID-19 [25], and has become overwhelmed by the years of the pandemic [26], this situation may have been responsible for the limited access of children and adolescents with asthma to outpatient services. This potential limitation could have contributed to the onset of asthma exacerbation [25,27] and its association with the increased severity of COVID-19 [15].

Cough is one of the body’s defense mechanisms against inhaled, aspirated, or locally produced substances, playing a crucial role in clearing the airways and maintaining patency [28]. It is the most common respiratory symptom in children with mild COVID-19 [29]. However, in this study, cough was associated with the severity of COVID-19. Viral infections, including COVID-19, are closely associated with autoimmunity and may heighten airway reactivity and trigger coughing. Furthermore, COVID-19 induces airway inflammation and mucus hypersecretion, potentially leading to a persistent, prolonged cough in children post-COVID-19, especially in those with allergies [29]. The study by Wongwathanavikrom et al. (2024) identified cough as a primary risk factor with the highest incidence of long COVID and abnormal lung function in children recovering from COVID-19 pneumonia [30].

The association between severity and the child’s overweight/obese nutritional status was borderline (*p* = 0.072), with a higher proportion of severe cases among overweight/obese individuals compared to non-severe cases. Obesity is related to a low-grade inflammatory state that compromises the entire body and the immune system and is considered an important risk factor for COVID-19 severity [31]. However, at the 5% significance level, it was not identified as a risk factor in this study. This may be attributed to the fact that this was a pediatric population where exposure to inflammatory markers may not have been sufficiently prolonged for the inflammatory processes to increase the risk of developing more severe COVID-19 [32], especially since none of the overweight/obese children presented any comorbidities associated with a worsening of obesity.

In the present study, hospitalized pediatric patients infected with SARS-CoV-2 were recruited not only from the COVID referral center, but also from a pediatric hospital. Despite efforts to follow the care flowcharts in the Specialized Healthcare Network proposed by the Ministry of Health [33] to minimize dissemination, the virus was not restricted to COVID referral centers. The fact that the study population was selected partly from a referral hospital did not constitute a study limitation, given that there were no statistically significant differences in the severity profile between the hospitals.

The requirement of a confirmatory laboratory diagnosis by RT-PCR for the inclusion of patients in the sample may have reduced the number of research participants since cases of COVID-19 with clinical and epidemiological confirmation were not counted. However, such rigor allowed the selection of a sample of pediatric patients who were effectively infected with SARS-CoV-2. Excluding patients with other comorbidities also allowed the analysis of only children in whom the severity could not be explained by anything other than SARS-CoV-2 infection.

## 5. Conclusions

Among pediatric patients hospitalized with COVID-19 during the study pandemic period, the association of clinical profile and case severity revealed a higher risk among infants aged under one year, those presenting with cough, and comorbidities of asthma and overweight/obese nutritional status. These findings underscore the importance of strengthening the healthcare network focused on pediatric care across all levels, training professionals for the adequate management and control of asthma, and monitoring nutritional status. This comprehensive approach is essential to optimize the care of children with this clinical profile when confronted with COVID-19 within the healthcare system.

## Figures and Tables

**Table 1:** Clinical characterization of children hospitalized with a diagnosis of COVID-19 (n = 191) between March 2020 and June 2021, Recife/PE, Brazil, 2024.

Cases of COVID-19	Number (%)
Age range	
Under 1 year	79 (41.4%)
From 1 to <5 years	61 (31.9%)
Over 5 years	51 (26.7%)
Sex	
Male	109 (57.1%)
Female	82 (42.9%)
Classification of COVID-19 cases	
Asymptomatic	7 (3.7%)
Mild	35 (18.3%)
Moderate	9 (4.7%)
Severe	111 (58.1%)
Critical	29 (15.2%)
Classified as severe	
Yes	140 (73.3%)
No	51 (26.7%)
Saturation < 95%	
Yes	89 (46.6%)
No	102 (53.4%)
Time of hospital stay: median (P25–P75)	5 (3–8)

**Table 2:** Association of severity in children diagnosed with COVID-19 (n = 191) in relation to biological characteristics, clinical characteristics, and signs and symptoms. Recife/PE, Brazil, 2024.

Characteristics	Severe Case (n = 140) Number (%)	Non-Severe Case (n = 51) Number (%)	OR (CI 95%)	*p*-Value
Age range				
Under 1 year	59 (42.1%)	20 (39.2%)	1.12 (0.50–2.48)	0.787
From 1 to <5 years	44 (31.4%)	17 (33.3%)	0.98 (0.43–2.25)	0.961
Over 5 years	37 (26.4%)	14 (27.5%)	Reference range	-
Sex				
Male	85 (60.7%)	24 (47.1%)	Reference range	-
Female	55 (39.3%)	27 (52.9%)	0.58 (0.30–1.10)	0.093
Residential region				
Recife	56 (40.0%)	23 (45.1%)	Reference range	-
Metropolitan Region of Recife	48 (34.3%)	20 (38.2%)	0.99 (0.48–2.01)	0.968
Other region	36 (25.7%)	8 (15.7%)	1.85 (0.75–4.58)	0.184
Nutritional status				
Adequate weight	115 (82.1%)	47 (92.2%)	Reference range	-
Overweight/obese	25 (17.9%)	4 (7.8%)	2.55 (0.84–7.74)	0.097
Comorbidity—Asthma				
No	83 (59.3%)	48 (94.1%)	Reference range	-
Yes	57 (40.7%)	3 (5.9%)	11.0 (3.26–37.0)	<0.000
Signs and symptoms				
Fever	95 (67.9%)	41 (80.4%)	0.51 (0.24–1.11)	0.094
Myalgia	2 (1.4%)	4 (7.8%)	0.17 (0.03–0.97)	0.046
Fatigue	11 (7.9%)	1 (2.0%)	4.26 (0.54–33.9)	0.170
Loss of appetite	10 (7.1%)	6 (11.8%)	0.57 (0.19–1.67)	0.312
Headache	6 (4.3%)	3 (5.8%)	0.72 (0.17–2.98)	0.646
Cough	108 (77.1%)	24 (47.1%)	3.79 (1.93–7.47)	<0.001
Runny nose	51 (36.4%)	17 (33.3%)	1.15 (0.58–2.25)	0.693
Nasal obstruction	19 (13.6%)	4 (7.8%)	1.85 (0.60–5.70)	0.288
Diarrhea	24 (17.1%)	13 (25.5%)	0.60 (0.28–1.30)	0.199
Abdominal pain	16 (11.4%)	10 (19.6%)	0.53 (0.22–1.25)	0.149
Vomiting	27 (19.3%)	13 (25.5%)	0.69 (0.33–1.49)	0.353
Dehydration	9 (6.4%)	4 (7.8%)	0.81 (0.24–2.75)	0.732

**Table 3:** Multivariate analysis of factors associated with severity in children diagnosed with COVID-19 (n = 191), Recife/PE, Brazil, 2024.

Characteristics	OR (CI 95%)	*p*-Value
Age range		
Under 1 year	3.23 (1.22–8.56)	0.018
From 1 to <5 years	1.09 (0.40–5.78)	0.859
Over 5 years	Reference range	-
Comorbidity—Asthma		
No	Reference range	-
Yes	13.2 (3.55–48.8)	<0.001
Cough		
No	Reference range	
Yes	2.72 (1.28–5.78)	0.009
Nutritional status		
Adequate weight	Reference range	
Overweight/obese	3.21 (0.90–11.4)	0.072

## Data Availability

The data presented in this study are available on request from the corresponding author due to the privacy of data.
